# Idiopathic Scrotal Calcinosis (ISC): A Familial Case Series of Two Cases

**DOI:** 10.7759/cureus.76711

**Published:** 2025-01-01

**Authors:** Pankaj Singodia, Deb Sanjay Nag, Radhika Narayan, Farah Rana, Manjeet Kumar

**Affiliations:** 1 Department of Plastic Surgery, Tata Main Hospital, Jamshedpur, IND; 2 Department of Anesthesiology, Tata Main Hospital, Jamshedpur, IND; 3 Department of Pathology, Tata Main Hospital, Jamshedpur, IND

**Keywords:** calcified globules, calcinosis, giant cell reaction, idiopathic, scrotum

## Abstract

Idiopathic scrotal calcinosis (ISC) is a rare condition characterized by the development of multiple, asymptomatic, painless nodules on the scrotal skin. While typically considered idiopathic, its etiology remains unclear, with potential links to the dystrophic calcification of pre-existing lesions. We present a familial case series of father and son with ISC, highlighting the clinical presentation, surgical management, and importance of histopathological confirmation.

## Introduction

Idiopathic scrotal calcinosis (ISC), also known as idiopathic calcinosis cutis of the scrotum (ICCS), is a relatively uncommon condition affecting predominantly men, though women can present with vulvar calcinosis. The condition involves the abnormal deposition of calcium in the scrotal skin or subcutaneous tissue. Clinically, ISC may be confused with other scrotal lesions, including calcified sebaceous cysts, steatocystomas, fibromas, atheromas, and xanthomas. Surgical excision with primary closure is the usual treatment, although extensive reconstruction may be necessary in severe cases.

## Case presentation

Case 1

**Figure 1:**
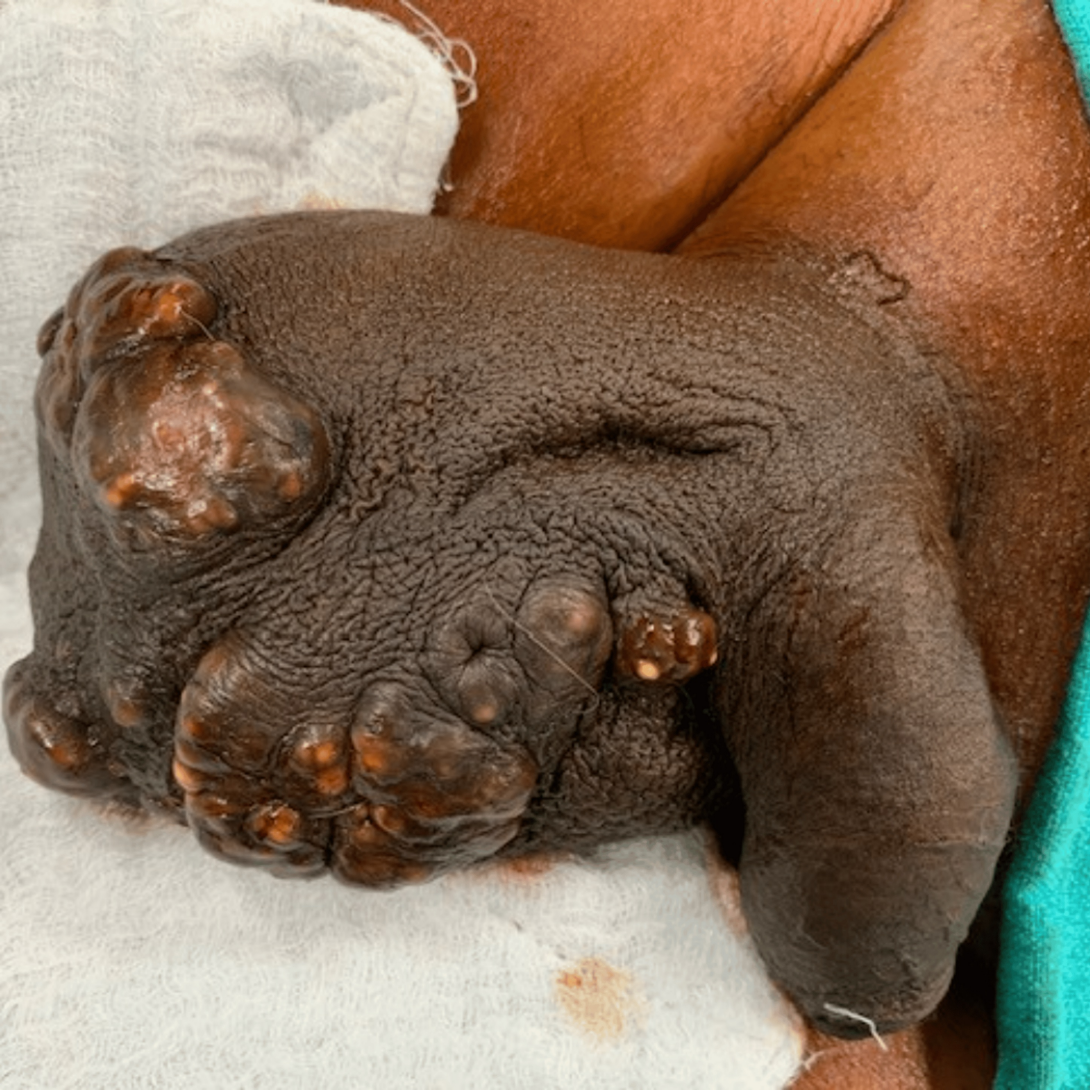
Multinodular lesions of the scrotal skin

A 55-year-old man presented with gradually enlarging, itchy, painless, yellowish-white scrotal nodules. The lesions, numbering several and up to 1-2 cm, were confined to the ventral scrotum, sparing the penis (Figure 1).

**Figure 2:**
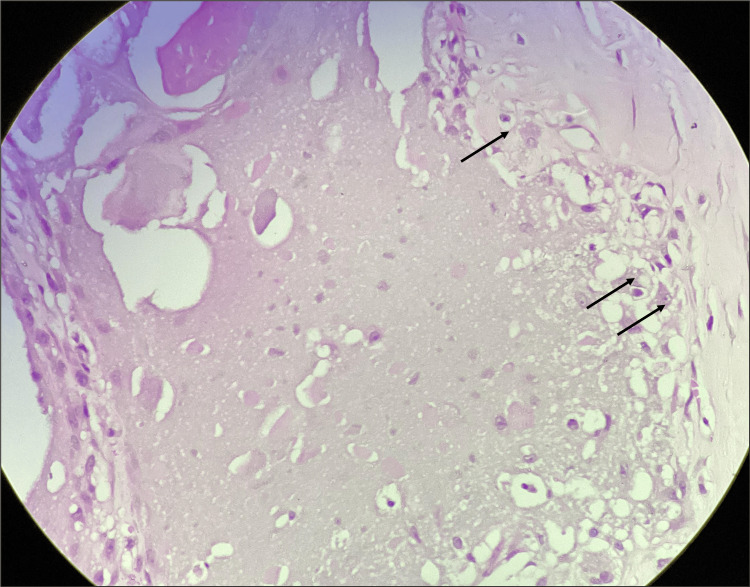
Calcium deposition with calcified globules consistent with idiopathic scrotal calcinosis Histiocytic response around calcified deposits (black arrows) (H&E, 200×; scale bar, 50 µm)

Physical examination revealed discrete swellings with chalky deposits. There was no history of trauma, sexually transmitted disease, or inflammation. There was no history of diabetes or immunosuppressant use, and there were no signs of hypercalcemia. The physical examination and review of systems were otherwise unremarkable. Laboratory tests, including serum calcium, phosphate, albumin, diabetes, and retroviral screening, were all negative. An excisional biopsy was performed under neuraxial anesthesia, followed by primary closure. Histopathology revealed normal squamous cell lining epithelium with large globules and circumscribed calcified material in the dermis. There were a foreign body giant cell reaction and few histiocytes around the calcified globules, confirming the diagnosis of scrotal calcinosis (Figure 2).

**Figure 3:**
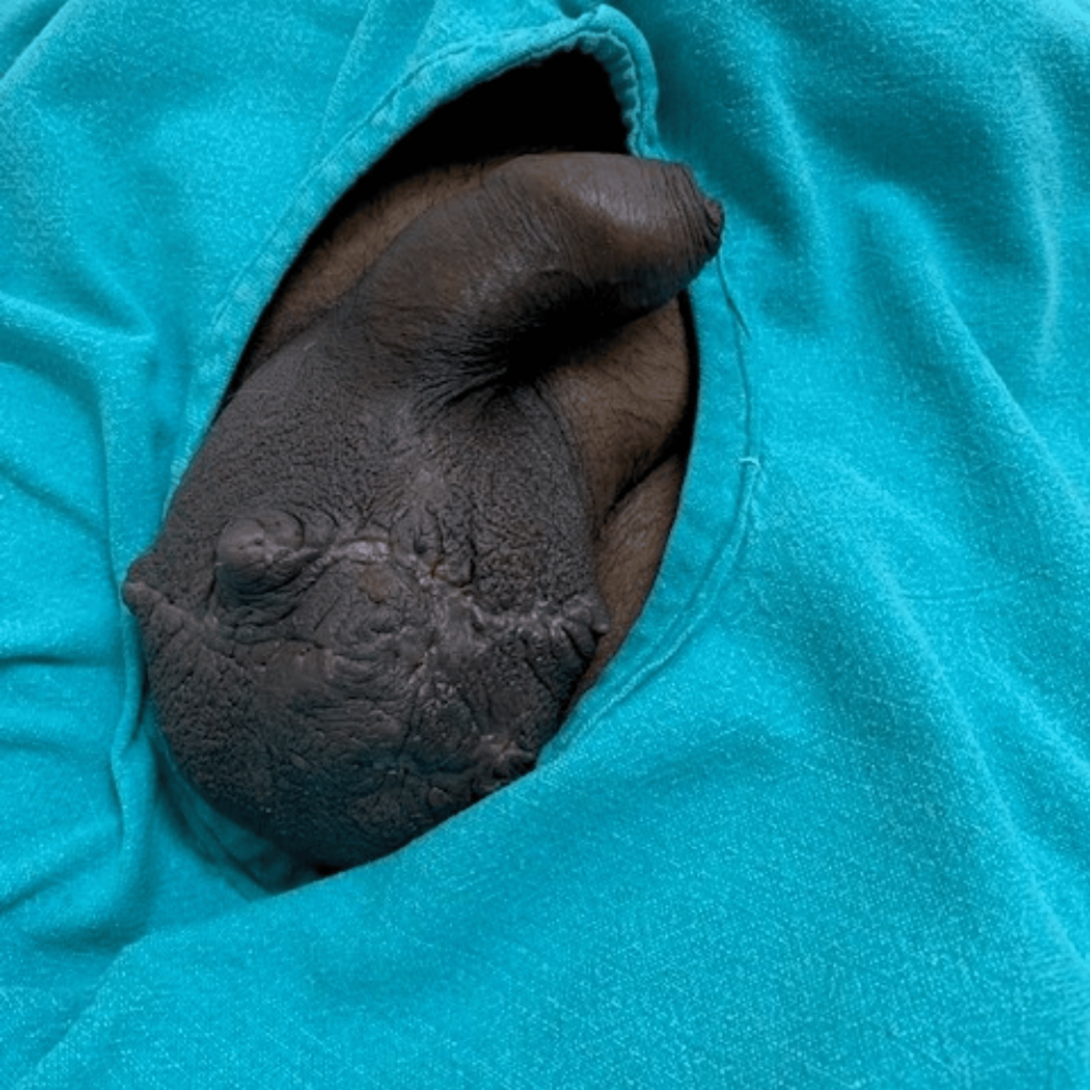
The reconstructed scrotum

Postoperatively, the patient had a good recovery with a healed surgical wound (Figure 3).

Case 2

**Figure 4:**
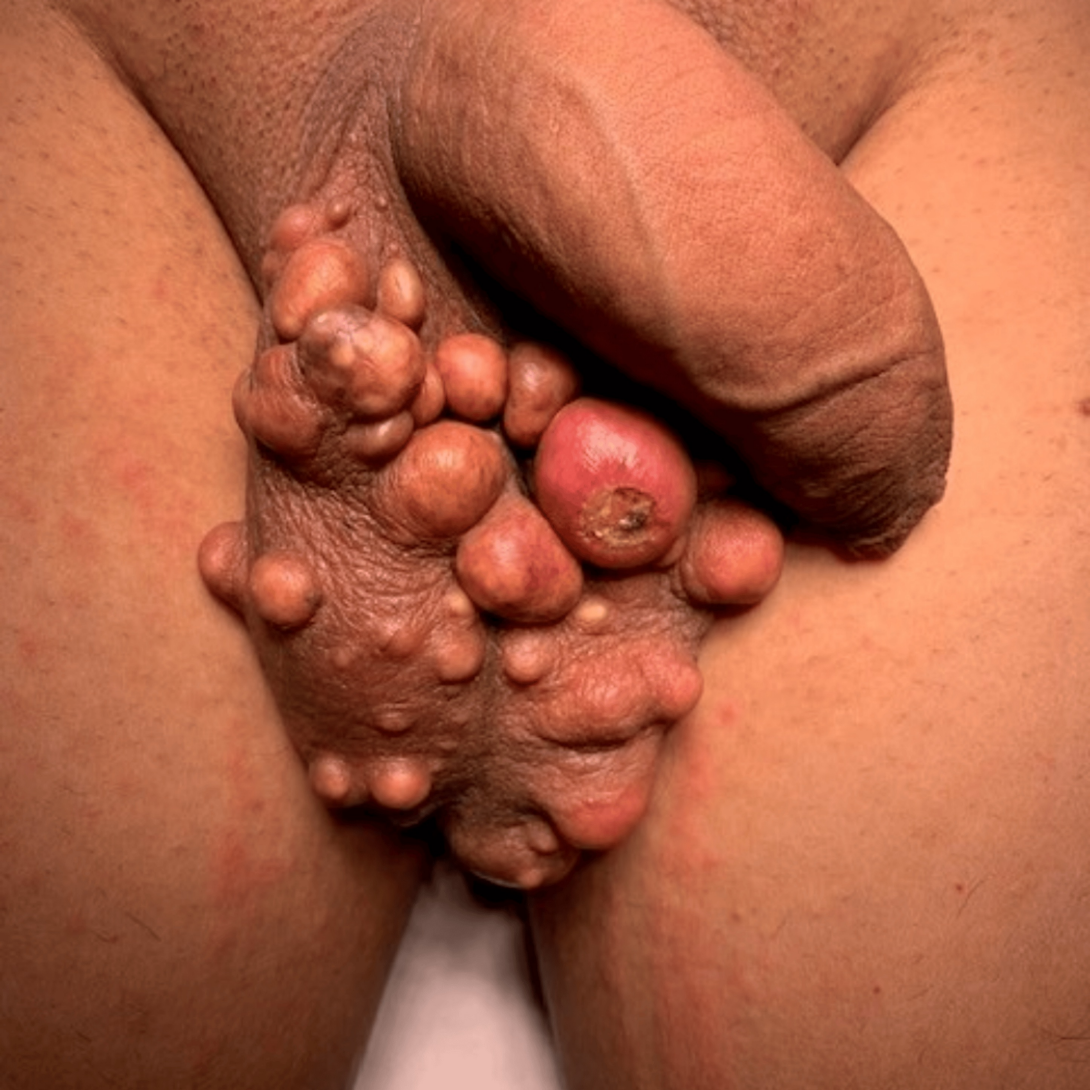
Multinodular lesions of the scrotal skin

Two years later, the patient's 27-year-old son presented with similar symptoms and findings of gradually enlarging, itchy, painless, yellowish-white scrotal nodules. The lesions were several and up to 1.5-2 cm (Figure 4).

**Figure 5:**
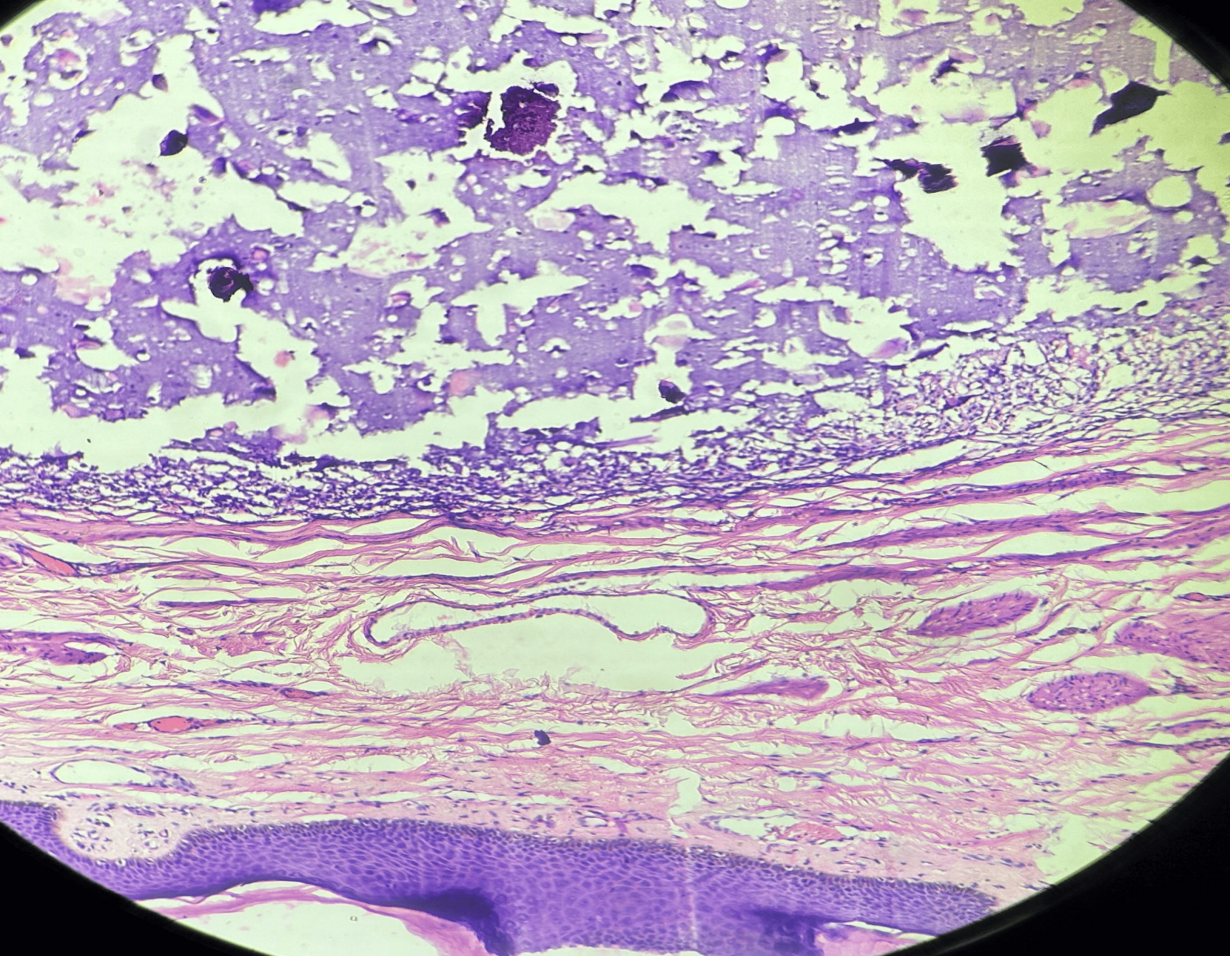
Subepithelial stroma shows variably sized dilated cyst spaces filled with basophilic calcium deposits consistent with idiopathic scrotal calcinosis (H&E, 100×; scale bar, 100 µm)

Under subarachnoid anesthesia, all scrotal nodules were surgically excised. The excised tissue included the affected skin and nodules, which extended to the dartos layer. After achieving hemostasis, the dartos layer and skin were meticulously sutured to reconstruct the scrotum. Serum calcium levels were normal in both patients. Histopathology revealed normal to focal irregularly hyperplastic squamous epithelial lining. Subepithelial stroma shows variably sized dilated cyst spaces filled with basophilic calcium deposits. The periphery of the cysts shows aggregates of stromal cells, chronic inflammatory cells, a few histiocytes, and some multinucleated giant cells. These findings are consistent with scrotal calcinosis (Figure 5).

**Figure 6:**
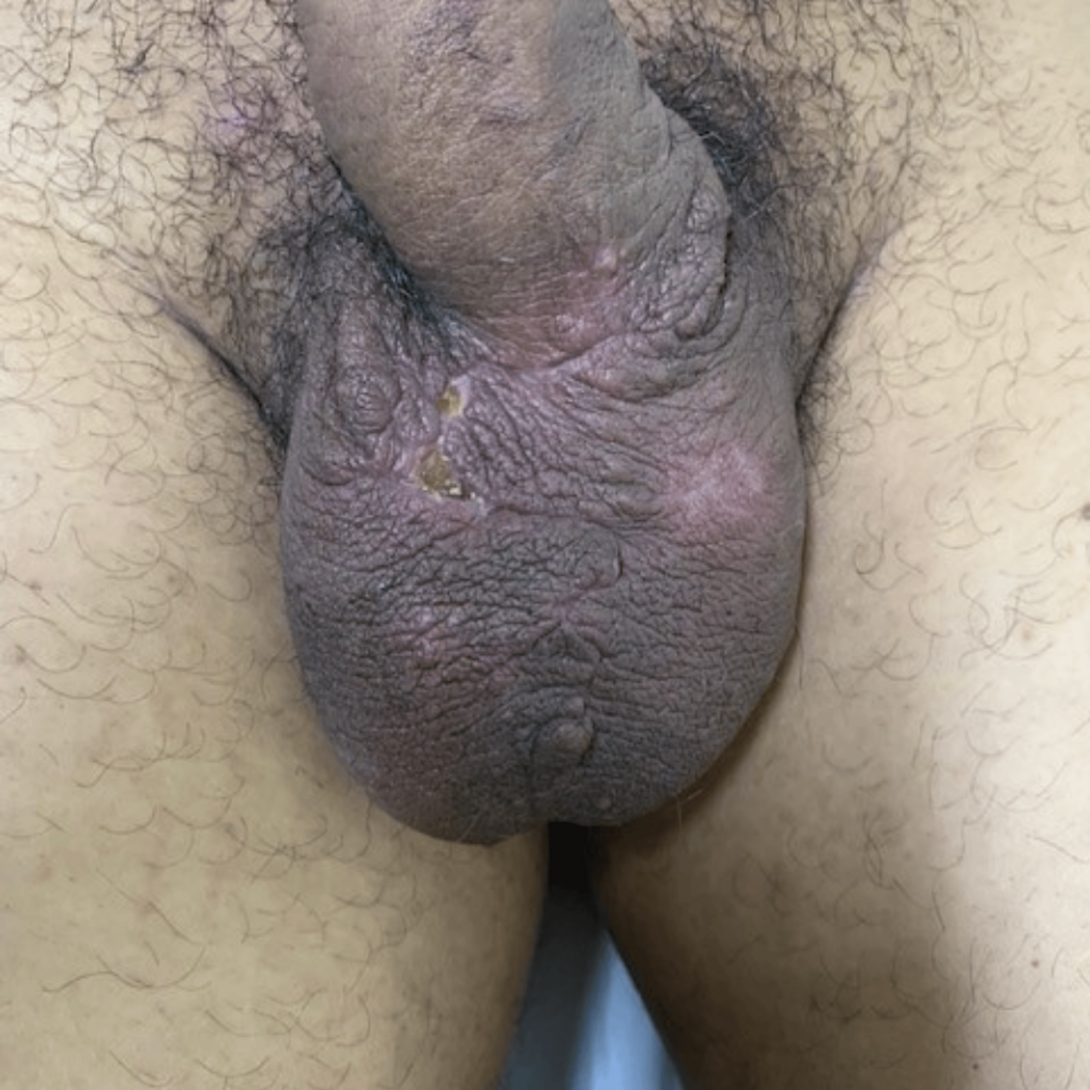
Postsurgical image after four weeks

The postoperative course was uneventful, and the cosmetic results were good (Figure 6).

The laboratory parameters of both cases were within normal limits, and there was no evidence of hypercalcemia in either of the two cases. It is summarized in Table 1.

**Table 1:** The laboratory parameters of the two cases

Laboratory Investigation	Case 1	Case 2	Normal Range
Hemoglobin	13.4 g/dL	14.2 g/dL	11.5-16.5 g/dL
Fasting Blood Glucose	96 mg/dL	83 mg/dL	70-100 mg/dL
Serum Creatinine	0.85 mg/dL	0.77 mg/dL	0.5-1.5 mg/dL
Serum Calcium	9.1 mg/dL	8.9 mg/dL	8.6-10.2 mg/dL
Serum Vitamin D	26.44 ng/mL	31.94 ng/mL	20-100 ng/mL
Serum Uric Acid	5.95 mg/dL	5.14 mg/dL	3.5-7.2 mg/dL

## Discussion

First described in 1883 [1], idiopathic scrotal calcinosis (ISC) is a rare, benign condition characterized by multiple calcified nodules in the scrotal skin without systemic calcium/phosphorus imbalance [2]. It typically presents in the third decade of life but can occur from ages nine to 85 years [3]. The etiology remains controversial, with some researchers suggesting that it results from the dystrophic calcification of epidermal cysts [4]. Diagnosis is confirmed through histological examination, revealing calcium deposits in the dermis [5]. Treatment is primarily surgical excision for cosmetic reasons [6]. Factors influencing treatment acceptance include impaired quality of life, reduced self-esteem, and the fear of sexual dysfunction [2]. While recurrence is uncommon, it has been reported in some cases [3]. ISC can be confused with other benign scrotal tumors, emphasizing the importance of proper diagnosis [7]. Differential diagnoses include calcified onchocercoma, neurofibroma, steatoma, lipoma, and fibroma. Histopathological examination is crucial for definitive diagnosis [8]. The pathogenesis of ISC remains unclear. While extra-skeletal calcification can be idiopathic, dystrophic, or metastatic, ISC typically occurs without metabolic abnormalities. Proposed etiologies include the dystrophic calcification of pre-existing lesions such as epidermal cysts, eccrine duct milia, or degenerated dartos muscle [9,10].

Surgical excision is the mainstay of treatment. The inherent laxity of the scrotal skin often allows for primary closure following excision, even with multiple lesions [11]. However, extensive disease may necessitate more complex reconstruction techniques, such as mesh grafting or flap procedures (e.g., groin flap and medial circumflex femoral perforator flap). Newer modalities of treatment using erbium-yttrium aluminum garnet (YAG) laser and carbon dioxide (CO_2_) laser are slowly evolving [12]. A series of five cases report superior outcomes with CO_2_ laser treatment as compared to conventional surgery [12]. However, the absence of a control group and the rarity of these cases limit the findings of the case series [12].

Although rare, with only about 200 cases reported by 2017, our familial case series of two cases of idiopathic scrotal calcinosis (ISC) is potentially the first of its kind [13]. Further research is needed to investigate a possible familial predisposition, but given the condition's rarity, additional familial case series would be needed to validate this etiology.

## Conclusions

ISC is a benign condition amenable to surgical excision and primary closure in many cases. However, the potential for recurrence necessitates thorough surgical removal and appropriate patient counselling. Accurate diagnosis through histopathological examination is crucial for optimal management. This familial case series underscores the importance of considering a familial predisposition in cases of ISC.
